# Diagnosis of a Liver Lymphangioma Using Contrast-Enhanced Ultrasonography (CEUS): Single Case Report

**DOI:** 10.3390/reports9010059

**Published:** 2026-02-13

**Authors:** Elīza Marta Budava, Ieva Pūce, Kalvis Kaļva, Nauris Zdanovskis

**Affiliations:** 1 Department of Radiology, Riga Stradins University, LV-1079 Riga, Latvia; kalvis.kalva@rsu.lv (K.K.);; 2 Department of Radiology, Riga East Clinical University Hospital, LV-1038 Riga, Latvia

**Keywords:** contrast-enhanced ultrasonography, liver lymphangioma, benign liver lesions

## Abstract

**Background and Clinical Significance**: CEUS enhances the visualization of vascular patterns within liver lesions, enabling differentiation between benign and malignant lesions, including hemangiomas, focal nodular hyperplasia, and hepatocellular carcinoma, with high accuracy. Lymphangiomas are rare benign lymphatic-system tumors, with intra-abdominal lymphangiomas accounting for approximately 5% of cases, most of which occur in the pediatric population. Intra-abdominal lymphangiomas commonly occur in multiple localizations due to lymphangiomatosis, but solitary lymphangiomas in adults are rare and easy to be misdiagnosed due to asymptomatic cases or non-specific symptoms. **Case Presentation**: A 65-year-old male with a history of left nephroadrenalectomy due to clear renal-cell carcinoma and paraaortic lymphadenectomy (staging pT3bN0M0V1R0) presented for a routine contrast-enhanced abdominal computer tomography examination. The scan showed several hypervascular structures that accumulate contrast in the arterial phase in the right liver lobe. Three years later, the patient developed complaints of abdominal pain and night sweats. Multiple MRI and CT examinations were performed, followed by a CEUS and a liver-core biopsy, which supported the diagnosis of hepatic lymphangioma. **Conclusions**: CEUS may be a more valuable evaluation method for follow-up examination than repeating CT and MRI scans. The real-time diagnostic possibility and tissue-perfusion data provide more profound information about the lesion of interest. Thus, it can be used as a primary diagnostic tool when a biopsy is performed. Although this method is relatively new, it can be applied in clinical settings with great value, and it saves time and resources.

## 1. Introduction and Clinical Significance

Contrast-enhanced ultrasound (CEUS) is a valuable imaging technique for the evaluation of focal liver lesions (FLL), especially in situations where conventional imaging methods, including B-mode ultrasound and color Doppler, fail to provide conclusive results. CEUS enhances the visualization of vascular patterns within liver lesions, enabling differentiation between benign- and malignant lesions, including hemangiomas, focal nodular hyperplasia (FNH), and hepatocellular carcinoma (HCC), with high accuracy [1,2]. Contrast agents, used in CEUS, are intravascular microbubble agents that are not excreted by the kidneys, making them particularly suitable for patients with impaired renal function.

According to the 2020 World Federation for Ultrasound in Medicine and Biology (WFUMB) guidelines, CEUS is recommended for further characterization of incidentally detected FLL identified on conventional ultrasound and in situations where contrast-enhanced computed tomography (CE-CT) or magnetic resonance imaging (CE-MRI) are contraindicated or yield inconclusive results. If a FLL cannot be visualized using a B-mode conventional ultrasound, CEUS is recommended to guide biopsy [3]. Rapid availability, an excellent safety profile, and high diagnostic accuracy for focal liver lesion characterization support the use of CEUS as a resource-efficient problem-solving imaging modality [3,4,5]. When CEUS does not allow confident lesion characterization, further evaluation with contrast-enhanced MRI or CT is recommended.

Lymphangiomas are rare benign lymphatic malformations whose etiology is usually due to the blockade of lymphatic reflux or abnormal lymphatic system development. It is characterized by the dilatation of lymphatic vessels in the liver parenchyma [6]. In 95% of lymphangioma diagnoses, localizations are in extra-abdominal regions—neck, head, and axilla—but intra-abdominal lymphangiomas are approximately 5% of cases and are most commonly encountered in the pediatric population [7]. Intra-abdominal lymphangiomas commonly occur in multiple localizations, including the spleen, liver, and mesentery, due to lymphangiomatosis [8], but solitary lymphangiomas in adults are rare and can be easily misdiagnosed due to asymptomatic cases or non-specific symptoms [9,10].

Solitary hepatic lymphangioma may mimic malignant liver formation; thus, being a rare finding, it can become misdiagnosed and lead to delayed diagnosis and, in some cases, even unnecessary surgical removal [6].

Current diagnostic algorithms for focal liver lesions are primarily designed around common benign- and malignant entities, while rare benign lesions such as solitary hepatic lymphangioma remain underrepresented in imaging-based decision pathways [3,8]. Because CEUS enables real-time assessment of microvascularization within cyst walls, septa, and solid components, it may provide decisive information in cystic- or multicystic lesions that otherwise remain indeterminate on a baseline ultrasound or even cross-sectional imaging [3,4,5].

## 2. Case Presentation

### 2.1. Clinical History

A 65-year-old male with a history of left nephroadrenalectomy due to clear renal-cell carcinoma and paraaortic lymphadenectomy with TNM staging pT3bN0M0V1R0, presented for a routine abdominal contrast-enhanced computer tomography (CE-CT) examination. The scan showed several hypervascular structures up to 1.5 cm that accumulated contrast in the arterial phase in the right liver lobe. In other phases, lesions were not visualized. No enlarged lymph nodes were visualized in the abdominal cavity or retroperitoneally, and no clinical symptoms were presented.

### 2.2. Imaging and Diagnosis

**Figure 1:**
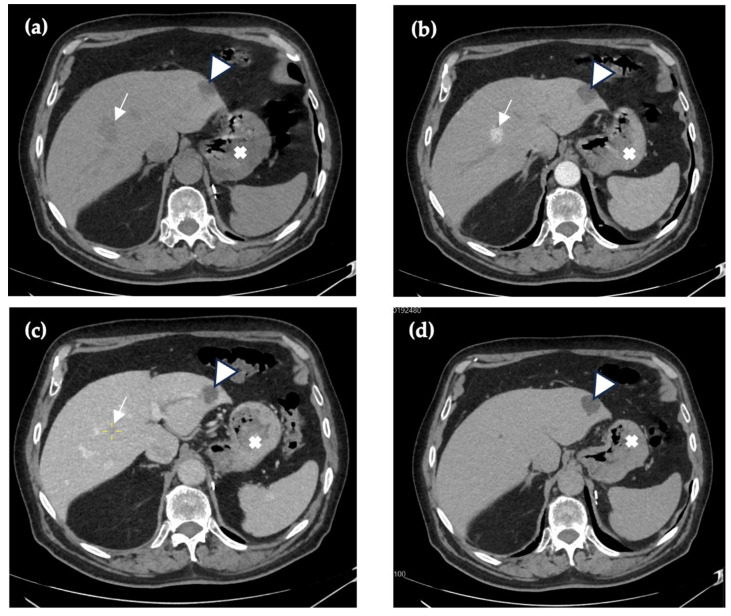
Thirty months after initial imaging, abdominal CE-CT shows a structure (arrow) in the liver with low attenuation consistent with fluid content in native phase (**a**), there are no infiltrative features or signs of surrounding parenchymal distortion in arterial phase (**b**) or no appreciable venous (**c**) enhancement within the lesion, and it merges with liver parenchyma in venous and 5 min delayed (**d**) phases accordingly. Additionally, a hypoattenuating hepatic cyst in segment II (triangle) and a renal cyst (cross) are visualized consistently throughout all CT phases.

Three years after the last imaging, the patient expressed that he had felt periodic pain in the abdomen for 9 months, which led to another follow-up contrast-enhanced computed tomography. The arterial phase showed hypervascular structures visualized in the arterial phase in the VII and VIII segments of the liver that had increased in size, up to 1.7 cm, without an increased size of the liver itself. New foci were found in the spleen—up to 1.0 cm. The contrast agent was not washed out in later phases, merging with the surrounding liver parenchyma. No changes in organ parenchymal density were detected. No lymphadenopathy was observed. In the porta hepatis, a few lymph nodes were visualized, with the largest measuring 7.3 mm (Figure 1). Blood tests for tumor markers were negative. A contrast-enhanced abdominal magnetic resonance imaging (CE-MRI) was scheduled.

**Figure 2:**
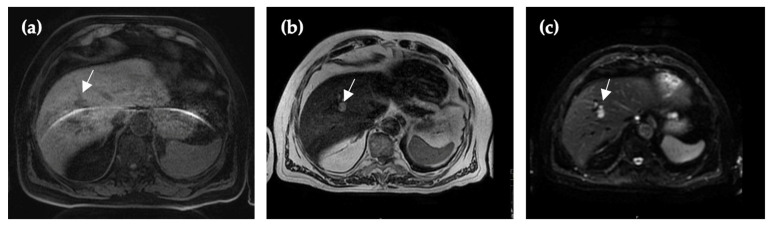
Thirty-two months after initial imaging, MRI reveals multiple hypointense lesions (arrow) in the VIII segment of the liver in T1 (**a**), in which no mural nodules or solid internal structures were identified. T2 (**b**) and DWI (**c**) sequences revealed hyperintense lesions with septa, which support the characteristics of lymphangioma.

CE-MRI revealed multiple hyperintense foci in the right liver lobe, up to 1.7 cm, and in the spleen (up to 1.1 cm). The structures were slightly limited in diffusion maps with high b-values (b = 1000), but no conclusive signs of malignancy were detected (Figure 2).

Over time, the patient continued to experience periodic pain in the abdomen and appeared to have periodic sweating during the night. Considering the clinical symptoms and previous examinations, fibrogastroscopy and fibrocolonoscopy were additionally performed. A polyp of the intestine was detected, and a routine polypectomy was performed. Otherwise, in these examinations, no other clinically significant findings were found.

**Figure 3:**
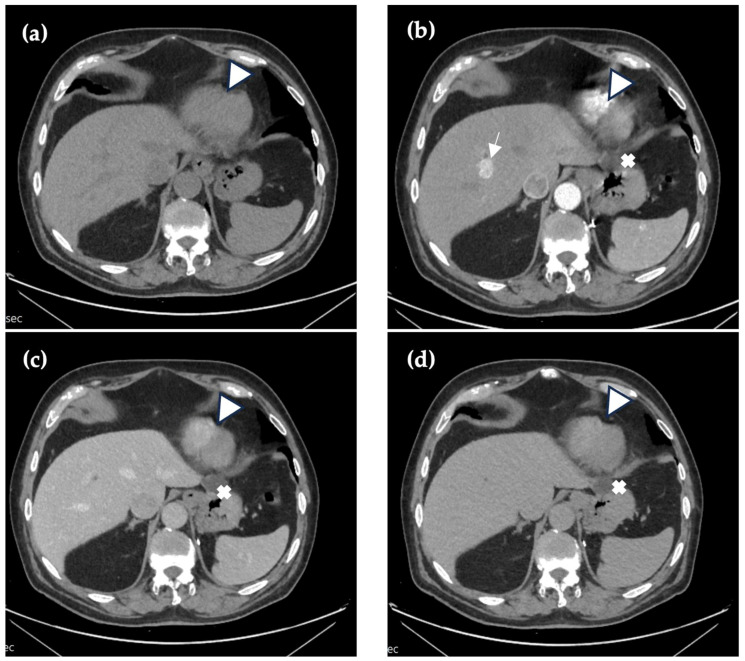
Forty-one months after first imaging, CE-CT revealed hyperdense lesions in the arterial phase (arrow) (**b**) with a slight increase in size, which can be characterized as a chylous component. In native (**a**), venous (**c**) and 5 min delayed (**d**) phases, the lesion demonstrates homogeneous hypoattenuation relative to the surrounding liver parenchyma. A hypoattenuating hepatic cyst in segment II (triangle) and a renal cyst (cross) can be visualized in native, arterial, venous, and 5 min delayed phases.

Forty-one months after the first CE-CT, a follow-up abdominal CE-CT scan was performed. The arterial phase in the right liver lobe revealed multiple hypervascular foci with a hypovascular central part in a few of them; however, in the venous and late phases, they appeared isodense (Figure 3). Compared with the previous abdominal CE-CT, the foci slightly increased in size, up to 1.8 cm. No new foci were detected in the liver. The hypervascular structures in the spleen remained the same size as previously, and no new nodules were detected. Enlarged lymph nodes were not visualized, with paraaortic lymph nodes measuring up to 0.5 cm. A CEUS scan and liver biopsy were scheduled.

**Figure 4:**
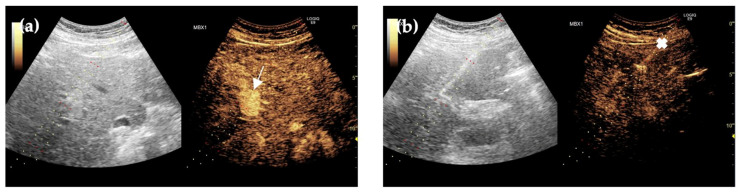
Forty-six months after first imaging, CEUS demonstrated contrast accumulation in the porto-venous phase (arrow) (**a**) and a biopsy needle (cross) positioned in the desired localization (**b**). On B-mode ultrasound, the lesion appears predominantly hypoechoic with possible internal thin septations.

Forty-six months after initial imaging, an abdominal ultrasound was performed, and no foci were detected. Following the CEUS scan, hypervascular formations were visualized close to the portal vein and liver arteries, while in the late phase, washout was not observed, indicating a benign lesion (Figure 4).

### 2.3. Biopsy and Final Diagnosis

Immediately after the CEUS scan, a liver biopsy was performed using local anesthesia with sol. Bupivacaine 5 mg/mL, 10 mL, in the right hypochondria. In total, 2.5 mL SonoVue contrast was administered intravenously to the patient, and a biopsy was performed with an 18 G × 20 cm biopsy device to obtain a sample of hypervascular structures. No free fluid was found around the liver after the procedure.

Pathohistological findings revealed preserved liver architecture, different sizes of lymph vessels with fibrous walls and fibrin fibers, and hemolyzed erythrocytes. Immunohistochemically, the biopsy material was CD10 negative, CD34 positive, CK7 negative, and PAX8 focally positive. No other specific lymphatic endothelial markers were used due to the rarity of lymphangioma and the lack of availability of markers such as D2-40, LYVE-1, and Prox-1.

The diagnosis of lymphangioma, in this case due to a lack of immunohistological markers, is based on the exclusion of differential diagnoses, such as other benign liver lesions. The absence of washout [5] in CEUS combined with long-term stability, histopathological absence of malignant cells, PAX-8 negativity, and CK negativity rules out metastasis or other malignancy [11]. Benign liver lesions, such as focal nodular hyperplasia, were excluded based on biopsy findings, which showed no evidence of nodular architecture, a central scar with radiating fibrous septa, or thick-walled arteries [11]. MRI characteristics in our case—T1 hypointense and T2 hyperintense with septa compared to FNH-isointense on T1 and isointense or slight hyperintensity on T2—narrows the differential diagnosis to two main possibilities: hemangioma or lymphangioma [12]. Hemangioma is excluded as a diagnosis based on imaging results, although in the arterial phase, it is typically discontinuous, nodular peripheral enhancement, or homogenous enhancement for small lesions, which is similar to the lesion in our case. In the portal-venous phase, CE-CT shows peripheral enhancement, while in our case, the lesion was iso-attenuating, without enhancement. MRI in case of hemangioma in T1 is hypointense relative to liver parenchyma, and T2 is hyperintense, but less than a hepatic cyst. Our case showed that this lesion was hypointense in T1 and hyperintense in T2, but with a chylous component and septations within the lesion, suggesting liver lymphangioma [13]. Hepatocellular adenoma is not considered due to its epidemiology—it is more common in women who use oral contraceptives, although it does not exclude the possibility of diagnosis. Lesions’ histological appearance—it has normal lobular architecture and normal hepatocyte structure in contrast to adenoma—“pseudo-capsule” and devoid of bile ducts [14].

Based on histological and immunohistochemical results, and diagnostics, the findings suggested the hypervascular structures to be lymphangiomas. No evidence of malignancy has been found.

Repeated biopsy was not possible due to the patient’s unwillingness to undergo an additional invasive procedure. Owing to the limited biopsy material and the absence of characteristic architectural features, definitive classification was not possible.

### 2.4. Follow-Up and Recommendations

Follow-up under the supervision of the patient’s family physician and urologist was recommended, with a contrast-enhanced CT examination planned at 12 months. This follow-up strategy was based on the patient’s oncological history and the associated risk of renal-cell carcinoma recurrence [15].

**Figure 5:**
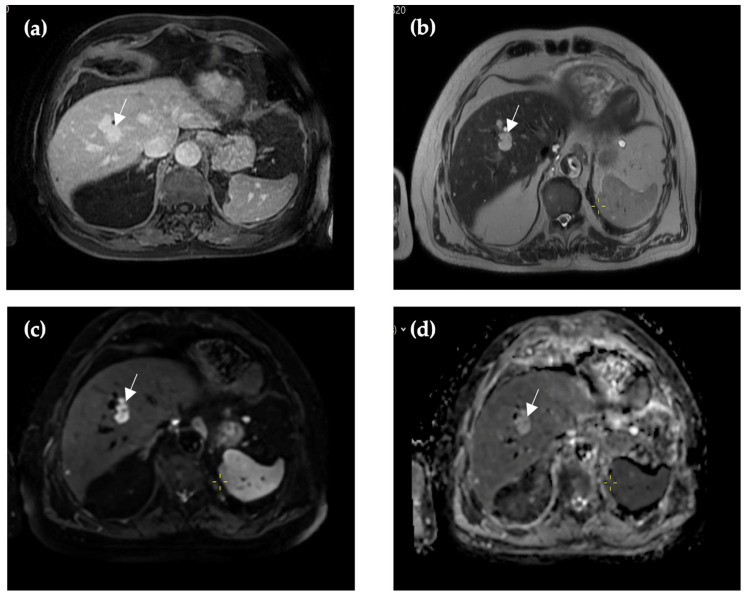
Sixty-four months after first imaging, CE-MRI shows liver lesions (arrow) without negative dynamics in T1 (**a**), T2 (**b**), DWI (**c**), ADC (**d**) sequences. Hyperintense signal in ADC and limited diffusion in high b-values in DWI refers to the possibility of a benign lesion.

The last follow-up imaging was made 64 months after the first CE-CT. A MRI detected that the largest lesion was 2.0 × 1.8 cm, without changes in size. Smaller lesions contrasted in the periphery, all of them with slight limitation of diffusion in the DWI sequence with high b-values (b = 1000) and hyperintense in ADC, which indicates the possibility of a benign lesion (Figure 5). No other new findings were detected. Hypervascular lesions in the spleen increased in size, up to 1.9 cm. Lymphadenopathy was not visualized, and no free fluid in the abdominal cavity was found.

## 3. Discussion

The pathogenesis of lymphangioma is still unknown, although there are theories. One of them includes the idea that lymphangiomas are due to abnormalities in embryogenesis, where the development of lymphatic tissue is impaired, causing abnormal regional lymphatic drainage, resulting in the dilatation of abnormal channels [16]. Other theories include trauma, inflammatory, and fibrotic processes, and also mechanical pressure and degeneration of lymph nodes [9,17]. Lymphangioma can be classified based on microscopic characteristics in five subtypes: capillary lymphangioma, cavernous lymphangioma, cystic lymphangioma or cystic hygroma, lymphangiohemangioma, and lymphangiosarcoma. Cystic is the most common type, which is caused by defects with communication between the venous and lymphatic systems, resulting in lymph accumulation, and is characterized by large cyst-like cavities filled with protein-rich fluid. The fluid component in lymphangioma could be detected. Depending on the anatomical site and complications caused by lymphangioma, there can be variations in the type of fluid; it can be serous or chylous, and due to hemorrhage, it could be described as coffee-like or, in case of infection, it could be purulent [18,19,20,21]. In this case, fibrin fibers and hemolyzed erythrocytes were found, which could be explained as a spontaneous or trauma-related hemorrhage.

About 40% abdominal lymphangiomas are asymptomatic and found accidentally on CT, MRI, or US [17]. Hepatic lymphangiomas, in most cases, have non-specific symptoms such as upper abdominal pain, which usually is due to the compression of surrounding tissue or organs [18]. In cases where lymphangioma causes intestinal obstruction, the patient may present with nausea and vomiting. In rare cases, there can be rupture of a tumor that can manifest as an acute abdominal pain [17]. In our case, the patient complained about periodic pain in the abdomen for 4 to 6 months. A year later, the patient experienced periodic excessive sweating in the night, which continued for at least 2 years.

Due to non-specific symptoms, the main diagnosis is based on imaging and biopsy. Usually on CT, MRI, and US, the structure can be seen as a simple or multiloculated cystic mass with internal septations. To differentiate the cause of the lesion, it is suggested to do a percutaneous biopsy. Due to complications, such as bleeding and malignant seeding, a biopsy may not be performed. In these cases, pathological examinations are made from resection material [9,16]. Non-specific features on imaging may promote differential diagnosis such as biliary cystadenoma, biliary cystadenocarcinoma, cystic metastasis, or even echinococcal cysts [15,16]. Cystic lymphangiomas are histologically present as a flat endothelial lining with lymphoid aggregates and smooth muscles in the wall, and in immunohistological testing, endothelial-lining cells react positively with CD31, CD34, and factor VIII-related antigen [22]. Differential diagnosis, such as biliary cystadenomas, in histology demonstrates cuboidal or columnar epithelium, supported by thick fibro-connective tissue and papillary folding into the cyst. To differentiate lymphangioma from biliary cystadenocarcinoma, histologically it will be seen proliferating cytologically malignant epithelium [22]. Unlike the present case, other studies have employed additional lymphatic endothelial antibody markers such as D2-40, LYVE-1, and Prox-1 to confirm the histopathological diagnosis and facilitate the differentiation of lymphatic endothelial cells [8].

CEUS is an advanced method for focal liver characterization compared to unenhanced ultrasound [5]. With unenhanced ultrasound, lesions can be identified by gray scale and Doppler, where easily characterizable lesions, such as an anechoic cyst or a homogeneous hyperechoic hemangioma, can be visualized. It is worth mentioning that often liver lesions are incompletely characterized and require further imaging and diagnostics [3]. CEUS’s main advantage is focal liver lesion characterization based on the enhancement pattern, and it has shown better detection of tumor vascularity compared to color Doppler or power Doppler [3]. To distinguish benign liver lesions from malignant ones, the CEUS late-phase has been used. Benign lesions can be seen with persistent enhancement with hypervascular or isovascular appearance compared to liver parenchyma, while malignant liver lesions appear with a washout and hypovascular appearance [5]. The main limitation of this method is that only one lesion can be visualized at a time [7]. Cysts and calcifications do not accumulate contrast agents; performing conventional US before CEUS is necessary to avoid misdiagnosis [3].

Most ultrasound contrast agents used for CEUS work intravascularly and, therefore, are considered a first-line contrast imaging method for patients with renal morbidities and for patients for whom contrast-enhanced CT and/or MRI are contraindicated. Ultrasound contrast agents do not cause harm if administered more than once in the same diagnostic procedure. Updated guidelines for CEUS in the liver strongly recommend CEUS as a first contrast imaging modality in patients with unclear FLL found in CT or MRI [3]. It should be noted that in this case, the indication for contrast-enhanced CT and MRI is independent of the presumed benign nature of specific lesions and is instead driven by the patient’s oncological history and risk of renal-cell carcinoma recurrence. This approach is recommended because renal-cell carcinoma metastases can present variably, and missed metastases due to inadequate imaging can impact clinical outcomes [15].

When a biopsy is mandatory for further diagnosis of the suspected lesions, a CEUS-guided biopsy shows increased accuracy and more accepted samples than conventional US-guided liver biopsies [23].

There is no single solution or therapy that can be applied for the treatment of lymphangioma; for example, surgery and sclerotherapy may be one of the treatment options for macrocystic lymphangioma patients, and there is increasing evidence about drug therapy based on altered signaling pathways found in structures affected by lymphatic malformation [24].

Currently, there is no standardized follow-up algorithm for hepatic lymphangioma. Published case reports describe imaging follow-up with stable outcomes; however, follow-up modality and timing vary considerably and are not standardized across studies [6,9].

## 4. Conclusions

Based on the number of radiological examinations performed and the prolonged uncertainty of the diagnosis of FLL, CEUS may be a more valuable evaluation method for follow-up examination than repeating CT and MRI scans. The real-time diagnostic possibility and tissue-perfusion data provide more profound information about the lesion of interest. Thus, it can be used as a primary diagnostic tool when a biopsy is performed. Although this method is relatively new, it can be applied in clinical settings with great value, and it saves time and resources.

## Data Availability

The original data presented in the study are included in the article, further inquiries can be directed to the corresponding authors.

## Abbreviations

The following abbreviations are used in this manuscript:

ADC	Apparent diffusion coefficient
CE-CT	Contrast-enhanced computed tomography
CE-MRI	Contrast-enhanced magnetic resonance imaging
CEUS	Contrast-enhanced ultrasonography
CT	Computed tomography
DWI	Diffusion-weighted imaging
FNH	Focal nodular hyperplasia
HCC	Hepatocellular carcinoma
MRI	Magnetic resonance imaging
WFUMB	World Federation for Ultrasound in Medicine and Biology
